# TXNIP shuttling: missing link between oxidative stress and inflammasome activation

**DOI:** 10.3389/fphys.2013.00050

**Published:** 2013-03-21

**Authors:** Troy Lane, Brenda Flam, Richard Lockey, Narasaiah Kolliputi

**Affiliations:** Division of Allergy and Immunology, Department of Internal Medicine, Morsani College of Medicine, University of South FloridaTampa, FL, USA

Thioredoxin-interacting protein (TXNIP) has been linked to cell apoptosis and inflammation in a number of diseases, including type 2 diabetes (Shah et al., 2013), atherosclerosis (Berk, 2007), and myocardial ischemia (Yoshioka et al., 2012). TXNIP has been identified as a tumor suppressor gene and in cancer, a loss of TXNIP can lead to cell proliferation (Zhou and Chng, 2013). The primary role of TXNIP is inhibition of thioredoxin (TRX), an important redox protein and promoter of cell growth. TRX is a ubiquitous protein that reduces thiols, especially insulin disulfides, and controls levels of reactive oxygen species (ROS) in cells, limiting damage from oxidative stress. Inhibition of TRX by TXNIP carries lethal consequences for cells and can promote destructive inflammation (Junn et al., 2000; Spindel et al., 2012). In human aortic endothelial cells with TXNIP knockdown, cultured under high glucose conditions to promote oxidative stress, there was a decrease in the amount of ROS generated as compared with control cells. This suggests that high levels of TXNIP inhibit the redox activity of cytoplasmic thioredoxin (TRX1) correlating with increased levels of ROS (Li et al., 2009). Mouse mesangial cells from wild type (C3H) and TXNIP-deficient mice (Hcb-19) were exposed to high glucose. After 3 h, ROS generation was 2.5–3 times greater in the cells from C3H compared to Hcb-19 (Shah et al., 2013). Mitochondrial thioredoxin, TRX2, like its cytoplasmic counterpart, TRX1, was found to regulate ROS and manage oxidative stress within mitochondria. HeLa cells were transiently transfected with a TRX2 expression vector and when treated with tumor necrosis factor alpha (TNF-a), a pro-inflammatory cytokine, generated approximately 50% less ROS than TNF-a treated control HeLa cells (Hansen et al., 2006). In this opinion piece, we interpret the facts on mitochondrial danger signaling: TXNIP's role in the mitochondria, interactions with mitochondrial TRX2 and activation of the NOD-like receptor protein 3 (NLRP3) inflammasome [an oligomeric complex activated by cellular infections or stress (Schroder and Tschopp, 2010)] signaling pathway not previously described.

While the role of TXNIP is defined, details of its localization within the cell remain unsolved. Based on previous research, which implicated TXNIP in mitochondrial death signaling, Saxena et al. explored the intracellular localization of TXNIP. They found that it is shuttled into mitochondria under oxidative stress while it is found in the nucleus under normal conditions (Saxena et al., 2010). TXNIP and TRX2 interactions were examined based on the parallel roles of cytoplasmic TRX1 and mitochondrial TRX2. Indeed, TXNIP and TRX2 are bound within the mitochondria, inhibiting the reductive power of TRX2, thus leaving ROS levels unchecked. The redox-regulated apoptosis-signal kinase (ASK1), a member of the mitogen-activated protein kinase family, has been deemed an important link between cellular stress and innate immunity (Barton and Medzhitov, 2003; Kolliputi and Waxman, 2009). ASK1 is usually bound to TRX2 under basal conditions, however, during stress and following TXNIP translocation to the mitochondria, ASK1-TRX2 binding is disrupted, triggering an apoptotic signal cascade. Unbound ASK1 is phosphorylated, signaling cytochrome C release and caspase-3 cleavage, eventually causing apoptosis (Bhattacharyya et al., 2003).

Zhou et al. showed that ROS causes TXNIP to associate with NLRP3 leading to inflammasome activation (Zhou et al., 2011). This study suggests that in the unstressed cell, TXNIP is bound to TRX1 and is inactive. In response to oxidative stress, ROS generation facilitates TRX1-TXNIP dissociation, thus increasing NLRP3-TXNIP association. Also, TXNIP translocates to the mitochondria, where it binds to TRX2 leading to mitochondrial dysfunction. However, the function of TRX2 in regulating the inflammasome has not been studied to determine whether it is an essential component of NLRP3 activation. The NLRP3 inflammasome, consists of NLRP3 oligomers and apoptosis-associated speck-like (ASC) adapter protein. The assembly of the NLRP3 inflammasome triggers caspase-1 activation leading to processing of interleukin-1ß (IL-1ß) (Schroder and Tschopp, 2010).

Studies by Shimada et al. and Saxena et al. elucidate the mystery of inflammasome activation that could hold a key to a therapeutic approach to limit unchecked cell apoptosis and inflammation with limited side effects. Shimada et al. reported that oxidized mitochondrial DNA (mtDNA) directly activates the NLRP3 inflammasome (Shimada et al., 2012). Their work showed that direct binding of oxidized mtDNA with NLRP3 serves as a trigger for inflammasome activation, indicating that apoptosis signaling leads to inflammasome activation and IL-1ß secretion. A key signaling point occurs when mtDNA reacts with ROS to become oxidized mtDNA, threatening imminent cell death (Shimada et al., 2012). Earlier, Nakahira et al. showed that mtDNA interacts with NLRP3 (Nakahira et al., 2011) but the exact mechanism is unknown. Significant cytosolic levels of free oxidized mtDNA could not be found in the absence of NLRP3, suggesting that it may stabilize mtDNA. As mentioned earlier, Saxena et al. described a novel mitochondrial apoptosis signaling cascade showing that TXNIP, under oxidative stress, translocates from the nucleus to mitochondria (Saxena et al., 2010). It is therefore tempting to speculate that mitochondrial shuttling of TXNIP may affect mitochondrial dysfunction and oxidation of mtDNA leading to NLRP3 inflammasome activation. Exploration of TXNIP's role in the mitochondria, interactions with mitochondrial TRX2 and mitochondrial danger signaling has shed light on a new NLRP3 signaling pathway not previously described.

**Figure 1:**
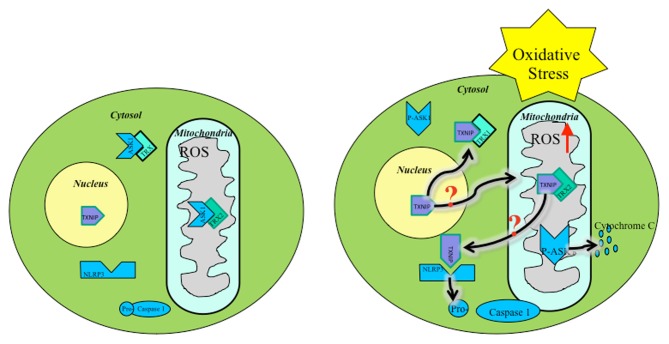
**Oxidative stress mediated TXNIP shuttling: TXNIP localization under basal conditions (Left), TXNIP rests in the nucleus.** TRX1-ASK1 binding in the cytosol and TRX2-ASK1 binding in the mitochondria maintains low ROS. Under oxidative stress (**Right**), TXNIP shuttles to the cytosol and mitochondria where it binds TRX1 and TRX2, respectively. ROS builds up, initiating mitochondrial distress signaling and eventual apoptotic cascade. It is unknown by what mechanism TXNIP shuttles to the mitochondria during oxidative stress.

ROS within mitochondria must initiate the mitochondrial cascade upstream, causing inflammasome activation. Several studies show that mitochondrial ROS are present prior to NLRP3 activation (Zhou et al., 2011). A possible source of ROS could stem from early TXNIP translocation following upstream cellular oxidative stress signals (Figure 1). Mitochondrial shuttling of TXNIP, followed by displacement of ASK1 by TXNIP-TRX2 binding, initiates downstream apoptotic signaling. Also, TXNIP inhibits TRX2 protection of mitochondria against ROS, allowing free oxidation of mtDNA, signaling distress, binding NLRP3, and triggering inflammasome activation. Downstream cleavage of caspase-3 by ASK1 leads to apoptosis serving as a secondary signal for NLRP3 activation.

Based on the mechanisms described, early apoptotic signals play a crucial role in inflammation. The earliest signal found is the shuttling of TXNIP from the nucleus to the mitochondria. A key therapeutic approach to limit inflammasome activation could be to inhibit mitochondrial shuttling of TXNIP. It was previously discussed that TXNIP inhibits reductive properties of TRX. In the mitochondria, this inhibition allows ROS to accumulate until TXNIP is shuttled to the cytoplasm where it interacts with NLRP3 (Davis and Ting, 2010). However, this mechanism needs to be defined. The increase in ROS causes the release of oxidized mtDNA, cytochrome C, and caspase-3 cleavage. Combined, these signals induce cell apoptosis and inflammasome activation, leading to IL-1ß secretion and localized inflammation. When TXNIP is in the mitochondria, it allows the accumulation of ROS. Therefore, finding and blocking the shuttling mechanism for TXNIP would allow TRX2 to maintain its reductive role and prevent downstream activation of NLRP3.

The role of TXNIP in inflammasome activation has proven invaluable in efforts to limit damage to cells and surrounding tissue. Once TXNIP reaches the mitochondria, it triggers a rapid cascade of apoptotic signals that end with TXNIP-NLRP3 binding and inflammasome activation. Early warning signals may be present prior to TXNIP translocation to mitochondria; however, such signals need to be identified. Further investigations into TXNIP localization and mitochondrial shuttling should be undertaken to completely map the signaling pathway, find factors that block TXNIP, and ultimately limit undesirable inflammasome activation.

In recent years, there is continued evidence of the central role of inflammasomes in sensing danger signals and orchestrating a subsequent inflammatory program (Kolliputi et al., 2010). However, due to the multitude of danger signals sensed by the NLRP3 inflammasome, it has remained a mystery how a single molecule can achieve this almost impossible task. A plausible explanation is that instead of detecting each danger signal individually, the NLRP3 inflammasome monitors the activity of the mitochondrion, which acts as an integrator of danger signals, including those of metabolic origin. Via a mechanism that remains elusive, excessive ROS production by mitochondria leads to activation of the inflammasome. Therefore, a clear understanding of this mechanism and regulation, will allow development of new therapeutic approaches to diseases involving the NLRP3 inflammasome.
